# Mechanical Behavior and Constitutive Model Characterization of Optically Clear Adhesive in Flexible Devices

**DOI:** 10.3390/mi13020301

**Published:** 2022-02-15

**Authors:** Yuexin Zhang, Shizhao Wang, Fang Dong, Yameng Sun, Can Sheng, Kun Ma, Zhiqiang Tian, Zhengfang Qian, Chingping Wong, Sheng Liu

**Affiliations:** 1 School of Power and Mechanical Engineering, Wuhan University, Wuhan 430072, China; zhangyuexin@whu.edu.cn (Y.Z.); wangshizhao@whu.edu.cn (S.W.); tianzhiqiang@whu.edu.cn (Z.T.); 2 The Institute of Technological Sciences, Wuhan University, Wuhan 430072, China; dongfang@whu.edu.cn (F.D.); sunyameng@whu.edu.cn (Y.S.); mk_175@whu.edu.cn (K.M.); 3 School of Mechanical Science and Engineering, Huazhong University of Science and Technology, Wuhan 430074, China; cansheng_chongqing@163.com; 4 College of Physics and Optoelectronic Engineering, Shenzhen University, Shenzhen 518060, China; zq001@szu.edu.cn; 5 School of Materials Science and Engineering, Georgia Institute of Technology, Atlanta, GA 30332, USA; cp.wong@mse.gatech.edu

**Keywords:** flexible devices, viscoelastic, hyperelastic, mechanical constitutive model

## Abstract

Optically clear adhesive (OCA) has been widely used in flexible devices, where wavy stripes that cause troublesome long-term reliability problems often occur. The complex mechanical behavior of OCA should be studied, as it is related to the aforementioned problems. Therefore, it is necessary to establish reasonable mechanical constitutive models for deformation and stress control. In this work, hyperelastic and viscoelastic mechanical tests were carried out systematically and relative constitutive models of OCA material were established. We found that temperature has a great influence on OCA’s mechanical properties. The stress and modulus both decreased rapidly as the temperature increased. In the static viscoelasticity test, the initial stress at 85 °C was only 12.6 kPa, 57.4% lower than the initial stress at 30 °C. However, in the dynamic test, the storage modulus monotonically decreased from 1666.3 MPa to 0.6628 MPa as the temperature rose, and the decline rate reached the maximum near the glass transition temperature (*T_g_* = 0 °C). The test data and constitutive models can be used as design references in the manufacturing process, as well as for product reliability evaluation.

## 1. Introduction

In the 5G era, special adhesive materials are generally used as interconnection structures to bond different function layers together, to work as a protective coating, or to realize device integration into a complete product [1,2]. Optically clear adhesive (OCA) has been widely used for cementing transparent optical elements, especially in photoelectric displays, due to its excellent optical performance, good bonding properties, and durable aging resistance [3]. Applications of flexible displays with short response times, wide viewing angles, and low power consumption have received considerable attention and have been regarded as a trend in future development [4,5,6,7,8]. OCA material is used for the bonding of film layers and also works as a stress buffer layer to improve bending performance [9,10]. The mechanical properties of OCA material will directly affect the design process and the evaluation of reliability. Therefore, it is essential to conduct a comprehensive mechanical behavior analysis and establish a reasonable material constitutive model before conducting design and rapid reliability assessments [11,12,13].

Yeh et al. focused on the bending stress analysis of foldable touch panels composed of seven-layer laminated structures. They analyzed the distribution of stress by applying four-point bending modeling, where the OCA material was simplified as a linear elastic material [14]. Salmon et al. modeled the folding of simplified display film stacks. They optimized the film stack through the bonding structure design of the OCA layer, where the viscoelastic properties were considered in the modeling process [15]. Ha et al. used OCA as buffer layers to generate multiple neutral planes for stress control, where OCA was also simplified as a linear elastic material [16]. Yunsik et al. conducted a finite element analysis and optimal design of a foldable display, during which only the hyperelastic properties were considered [17]. Cheng et al. used FEA tools to establish a simulation model of a flexible display and analyzed the thin-film stacks. The mechanical behavior was described by using the traction–separation law to obtain the relative material parameters [18]. Nishimura et al. used folding stiffness measurements and FEM simulation to establish the relationship between the position of the split neutral layer and folding stiffness, with OCA material simplified as a linear elastic material [19]. Ma et al. characterized the viscoelastic properties material with a stress relaxation test in the process of OLED bending modeling and simulation [20]. In fact, stress relaxation had already occurred in the process of displacement loading, meaning that the regular static method could not accurately characterize the viscoelastic properties.

OCA materials have both the characteristics of hyperelasticity and viscoelasticity as a type of high-molecular polymer. Their mechanical properties are greatly affected by environmental factors such as temperature change and moisture absorption. The flexible panels continuously experience cyclical deformation and continuous dynamic transformation while in service. As the bonding material of all film layers, OCA material consistently experiences repeated large deformation behaviors at a certain frequency and is constantly affected by ambient vibration and temperature changes. The OCA material plays a crucial role in product reliability and the service life of flexible devices. Therefore, providing a good description of mechanical properties is an important issue.

Constitutive models are the basis of formulating some more elaborate constitutive equations, such as damage models [21]. In addition, numerical simulation has been regarded as an important method to aid design. The accuracy of constitutive models has an important influence on mechanical analysis and calculation accuracy. Reasonable constitutive models can make a simulation’s results more reliable. Therefore, it is particularly important to propose models that can accurately characterize mechanical properties and that can be easily applied to finite element analysis software.

This work describes comprehensive mechanical experiments on OCA materials by using a universal testing machine and a Dynamic Mechanical Analyzer (DMA). The results of the hyperelasticity experiments were analyzed and fitted using the Mooney–Rivlin, Yeoh, and Ogden models based on phenomenological theory. Following this, the fitting results of different models were compared. Quasi-static and dynamic experiments on viscoelasticity were conducted using DMA, the generalized Maxwell model was used to describe the experimental data, then the material parameters related to viscoelasticity were obtained. In addition, the macroscopic mechanical performance was explained at the molecular level to some extent, which has a certain significance for helping to understand the complex mechanical behavior.

## 2. Constitutive Model

### 2.1. Hyperelastic Constitutive Models

OCA material is characterized by a very high deformability and almost complete recoverability after deformation. Hyperelastic constitutive models can be constructed to characterize the mechanical behavior of OCA material. There are two efficient approaches used to describe mechanical behavior: molecular-chain-based models and the phenomenological theory [22].

In this work, the Mooney–Rivlin, Yeoh, and Ogden models based on phenomenological theory were used to characterize the hyperelastic properties of OCA material. A hyperelastic material is an ideally elastic material for which the stress–strain relationship derives from a strain–energy density function. An infinite convergent power series of the invariants of the Green deformation tensor can be expressed as [23]:

(1) $$W=\overset{N}{\underset{i+j=1}{\sum }}C_{ij}{\left(I_{1}-3\right)}^{i}{\left(I_{2}-3\right)}^{j}+\overset{N}{\underset{k=1}{\sum }}\frac{1}{D_{k}}{\left(I_{3}^{2}-1\right)}^{2k}$$

where the *C_ij_* is the material characteristic constant. *D_k_* is the constant of the material compressibility. *N* is the number of terms in the equation. *I_i_* (*i* = 1, 2, 3) are principal invariants of Cauchy–Green deformation tensor given by:

(2) $$I_{1}=\lambda_{1}^{2}+\lambda_{2}^{2}+\lambda_{3}^{2}$$

(3) $$I_{2}=\lambda_{1}^{2}\lambda_{2}^{2}+\lambda_{2}^{2}\lambda_{3}^{2}+\lambda_{1}^{2}\lambda_{3}^{2}$$

(4) $$I_{3}=\lambda_{1}^{2}\lambda_{2}^{2}\lambda_{3}^{2}$$

where $\lambda_{i}$ (*i* = 1, 2, 3) are stretches of the material in the principal directions.

*I*_3_ does not contribute to the strain energy because of the incompressible property and W depends on only two independent deformation invariants; thus, the function can be reasonably simplified. The stress–stretch relation can be written as follows:

(5) $$\sigma =2\left[\lambda^{2}-\frac{1}{\lambda }\right]\cdot \left[\frac{\partial W}{\partial I_{1}}+\frac{1}{\lambda }\frac{\partial W}{\partial I_{2}}\right]$$

where $\varepsilon =\lambda -1,\lambda_{1}=\lambda_{2}=1/\sqrt{\lambda_{3}}=1/\sqrt{\lambda }$ (which are valid for tensile tests).

The Mooney–Rivlin constitutive model is a special form of polynomial model and expressed by functions of *I*_1_ and *I*_2_, which can be used for fitting the medium deformation stages of materials. For the Mooney–Rivlin model, the three-parameter and five-parameter strain–energy density function can be respectively expressed as [24]:

(6) $$W=C_{10}\left(I_{1}-3\right)+C_{01}\left(I_{2}-3\right)$$

(7) $$W=C_{10}\left(I_{1}-3\right)+C_{01}\left(I_{2}-3\right)+C_{11}\left(I_{1}-3\right)\left(I_{2}-3\right)+C_{20}{\left(I_{1}-3\right)}^{2}\;+C_{02}{\left(I_{2}-3\right)}^{2}$$

where *C*_10,_ *C*_01,_ *C*_11,_ *C*_20,_ and *C*_02_ are the material constants.

The Yeoh model [25,26,27] can generate a typical S-shaped stress–strain curve, which can be used to characterize the experimental data under large deformation conditions, especially in the hardening stage. The strain energy function of the Yeoh model is as follows:

(8) $$W=C_{10}\left(I_{1}-3\right)+C_{20}{\left(I_{1}-3\right)}^{2}+C_{30}{\left(I_{1}-3\right)}^{3}$$

in which *C*_10,_ *C*_20,_ and *C*_30_ are material constants and $I_{1}$ is the first strain invariant.

The Ogden model [28] is more flexible: the 3rd-order model can fit the mechanical behavior well and accurately represent the large-scale deformation, in general. The strain energy can be expressed as a function of the principal stretches:

(9) $$W=\sum_{i=1}^{N}\frac{\mu_{i}}{\alpha_{i}^{}}(\lambda_{1}^{\alpha_{i}}+\lambda_{2}^{\alpha_{i}}+\lambda_{3}^{\alpha_{i}}-3)$$

where the coefficient *α_i_* is a real number, either positive or negative. *µ_i_* is a material constant.

The force–displacement data directly measured by the uniaxial tensile test were processed to obtain engineering stress–strain data, which were used for constitutive model fitting.

### 2.2. Viscoelastic Constitutive Models

OCA material has typical viscoelastic mechanical characteristics such as stress relaxation and creep, which can be reasonably characterized by establishing a viscoelastic constitutive model.

In this work, the static and dynamic viscoelastic mechanical behaviors were tested to analyze viscoelastic properties accurately. Hook’s spring and the basic dashpot element were used as basic elements to represent the ideal elastic solid and viscous fluid, respectively. Two basic elements were combined in different forms to describe the complex viscoelastic mechanical properties. At present, widely used viscoelastic constitutive models [29,30] include the Maxwell, Kelvin, three-parameter, and generalized Maxwell models. We selected the appropriate constitutive model according to the specific engineering situation.

**Figure 1:**
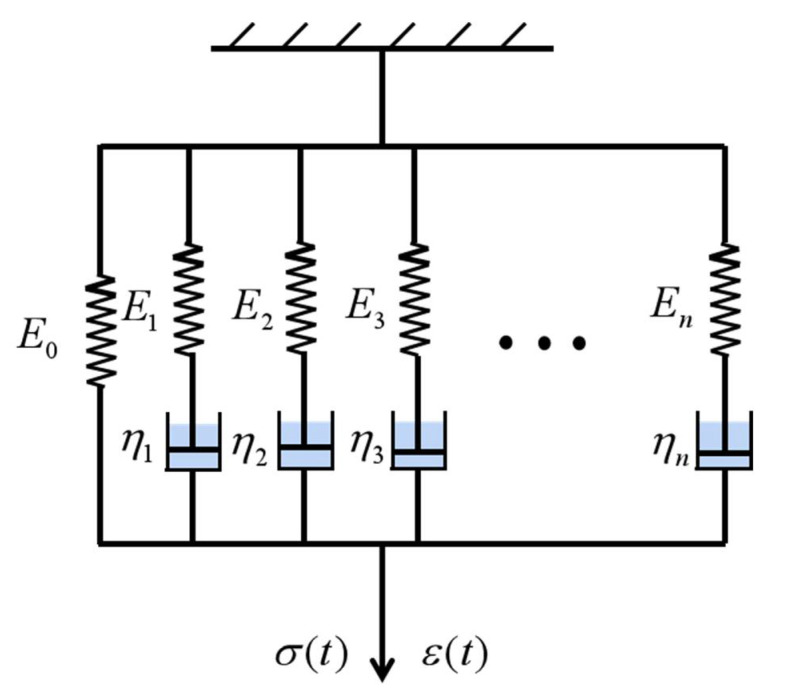
Generalized Maxwell model.

The generalized Maxwell model connects several Maxwell elements and one spring element in a parallel connection, as shown in Figure 1, to simulate relaxation behavior. In this work, the generalized Maxwell model was selected. When the loading strain $\varepsilon (t)=\varepsilon_{0}$ is maintained, the expression for the stress of the basic Maxwell model can be obtained as [31]:

(10) $$\sigma \left(t\right)=\varepsilon_{0}Ee^{-\frac{t}{\tau }}$$

where τ=ηE is the relaxation time coefficient of the basic Maxwell model.

For the generalized Maxwell model, the total relaxation modulus can be obtained by single-component superposition, and the expression can be obtained as follows:

(11) $$E\left(t\right)=E_{\infty }+\overset{N}{\underset{i=1}{\sum }}E_{i}\cdot e^{-\frac{t}{\tau_{i}}}$$

where *E(t)* is the relaxation modulus of the generalized Maxwell model, *E*_∞_ is the long term (tensile) modulus, and *E_i_* is the elastic modulus in the *i*th basic Maxwell element (*i* = 1, 2, 3…, *n*). The normalized modulus, *g*_i,_ is generally used as the dimensionless expression of the material constant and the groups of *g_i_* and *τ_i_* are called the Prony series, which can be used for the material definition and model simulation. The expression can be obtained as follows:

(12) $$g(t)=1-\sum_{i=1}^{N}g_{i}(1-e^{-\frac{t}{\tau_{i}}})$$

According to the Boltzmann superposition principle, the stress response of the generalized Maxwell model can be expressed as:

(13) $$\sigma (t)=\varepsilon_{0}E(t)+\int_{0}^{t}E(t-\xi )\frac{d\varepsilon (\xi )}{d\xi }d\xi$$

where *σ(t)* is the total stress response, *t* is the current time, and *ξ* is the delay time.

After testing the mechanical behavior of stress relaxation and dynamic viscoelasticity, the generalized Maxwell model was used to describe the test data, and the material parameters related to viscoelasticity were obtained.

## 3. Materials and Methods

### 3.1. Sample Preparation

**Figure 2:**
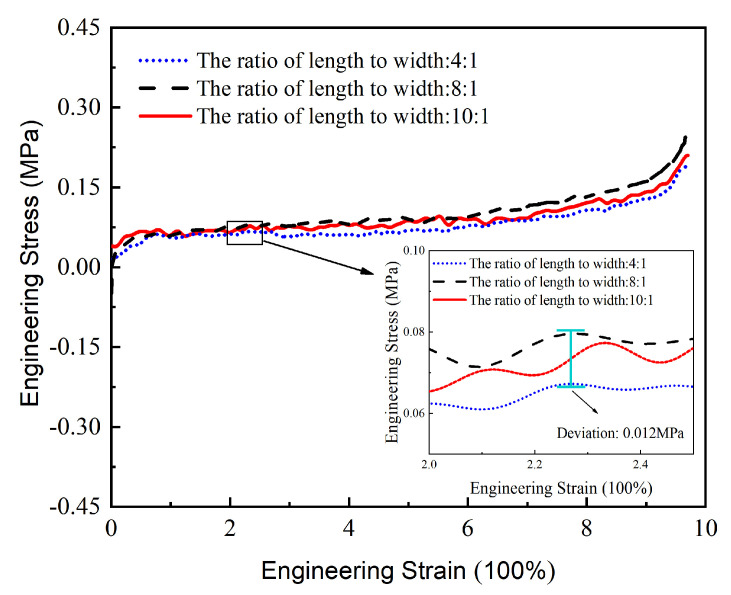
Stress–strain data of samples with different length–width ratios.

At present, there are no uniform sample preparation standards for OCA materials. Therefore, preliminary tests were carried out to study the influence of sample size on their mechanical behavior, where the ratio of length to width was different. The parameters of the test samples are shown in Table 1. Samples with different length–width ratios were used for a uniaxial tensile test at a constant tensile rate. The stress–strain data measured in the experiment are shown in Figure 2.

The range and variation trends of test data with different length–width ratios were basically the same when the tensile rate was fixed at 100 mm/min and the temperature was 30 °C. We found that the deviation caused by sample size was within the range of 10% (Figure 2), which shows that the material properties are scale-independent. To meet the requirements of the testing equipment, a sample with a size of 40 mm × 10 mm × 1 mm was selected for subsequent experiments.

### 3.2. Experimental Procedure

#### 3.2.1. Hyperelastic Experiment

**Figure 3:**
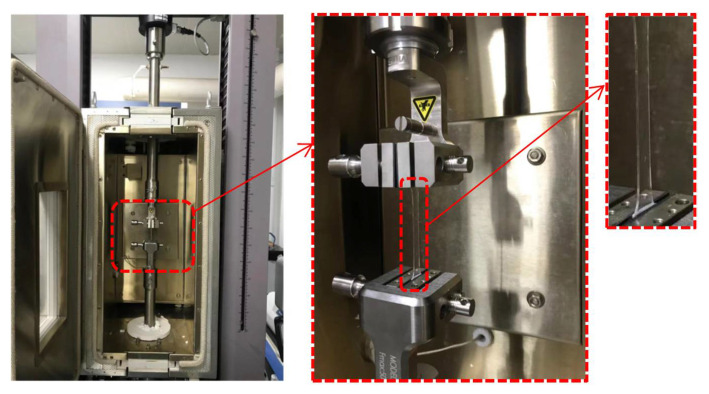
Tensile testing machine of the OCA material.

In our study, to characterize the hyperelasticity of OCA material, film samples were prepared and a universal testing machine was applied. Uniaxial tensile tests were conducted under four different tensile rates at room temperature. Figure 3 shows both the testing machine and a stretched sample.

#### 3.2.2. Viscoelastic Experiment

**Figure 4:**
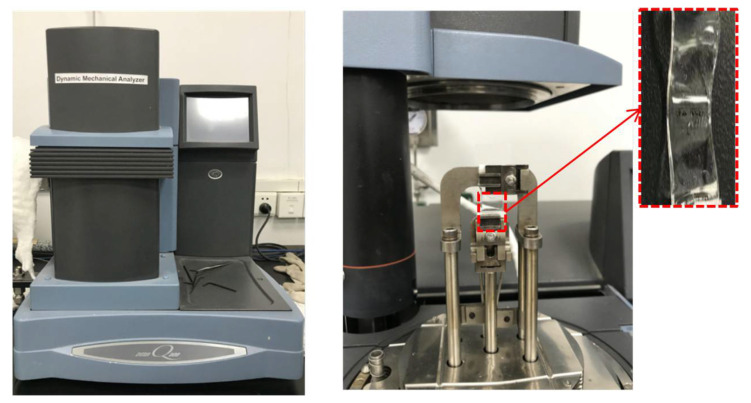
Tensile test device of DMA.

In this work, the OCA material analysis was performed in film tension mode using a Dynamic Mechanical Analyzer (DMA, TA Q800, New Castle, DE, USA) to characterize the static and dynamic viscoelasticity. The DMA machine and the sample are shown in Figure 4.

Usually, static viscoelastic tests include stress relaxation and creep. The static parameters can be obtained by a stress relaxation test. To evaluate the static viscoelastic properties, uniaxial stress relaxation tests at 30 °C, 60 °C, and 85 °C were carried out for three specimens in each group. The changes in stress and relaxation modulus over time were obtained and then used for the parameter acquirement of the viscoelastic constitution model. However, the test data could not accurately describe the relaxation process because stress relaxation had already occurred during the process of displacement loading. Therefore, it was necessary to carry out dynamic viscoelastic tests on OCA materials to obtain the viscoelastic parameters truly and accurately.

The dynamic viscoelastic tests were carried out using the DMA Q800, where a 0.01 N preload force and a 125% force track were applied. The force track adjusted the total force to avoid the sample automatically buckling during the test. The amplitude of the sinusoidal force applied to the samples was set at 20 μm. Then, the viscoelastic properties were measured in the multi-temperature mode, where the temperature changing rate was set to 3 °C/min and ranged from −40 °C to 125 °C. The range was selected to refer to the J-STD-020D standards. The sinusoidal force frequencies were set at 1 Hz, 2 Hz, 5 Hz, and 10 Hz, respectively. Finally, the data were processed to establish the viscoelastic model.

## 4. Results and Discussion

### 4.1. Hyperelasticity of OCA Material

The universal testing machine was used to conduct uniaxial tensile experiment at different strain rates. The force and displacement data were recorded by the data acquisition system and used to calculate the corresponding engineering stress, *σ_T_*, and strain, *ε_T_*, as follows:

(14) $$\varepsilon_{T}=\frac{l-l_{0}}{l_{0}},\;\sigma_{T}=\frac{F}{bh}$$

where *l*_0_ is the original length of the sample, *l* is the stretched length, *F* is the tensile load, and *bh* is the cross-section area.

**Figure 5:**
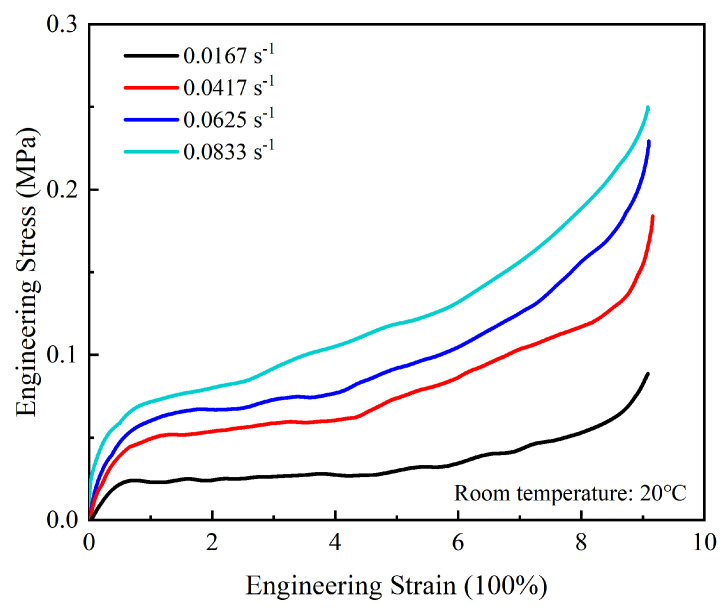
Tensile stress–strain curves for various strain rates.

The stress–strain curves of OCA material at different tensile rates are shown in Figure 5. It can be clearly seen that all the engineering stress–strain curves show S-shape non–linear characteristics and exhibit strain hardening and strain-rate hardening effects. The stress–strain curves indicate three deformation stages in the entire tensile process. The stress–strain curve is approximately linear at the beginning of the stretching process. The initial region represents the linear–elastic stage, during which the stress is proportional to the strain and the relationship satisfies Hooke’s law. As the stretching continues, the stress increases slowly with a small slope as the strain increases that represents a highly elastic stage in the 2nd region. After that, the OCA material enhances the ability to resist deformation, meaning that more stress is needed to produce the same strain, exhibiting a significant strain hardening effect. We can also observe that the elastic modulus increases with the increase in the strain rate; it increases about 3.89 times as the strain rates increases to 0.0833 s^−1^ in this work.

**Figure 6:**
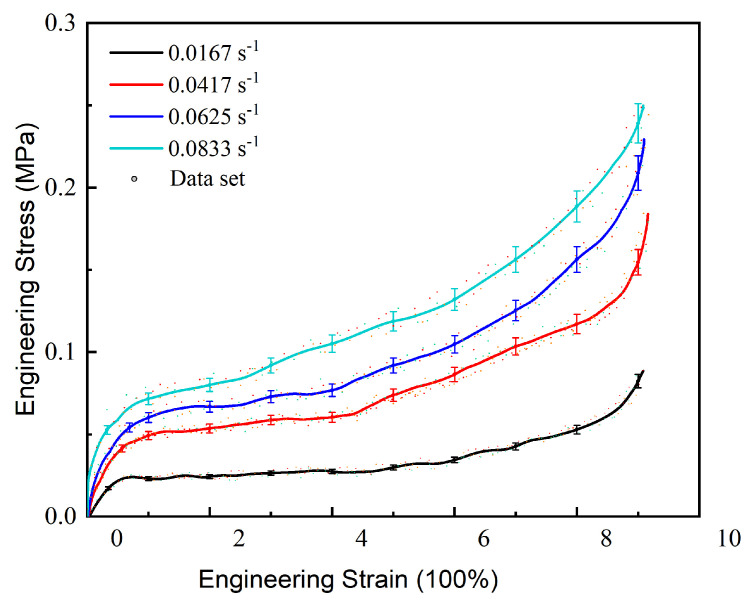
Error range analysis by repeated experiments.

Repeated tests under the same tensile condition were carried out to verify the errors, and Figure 6 shows the error range at different strain rates. The dataset includes the experimental data of three samples tested separately. It can be clearly seen that these tests have consistent trends and that the error is approximately 5%, proving that the experiments have a good repeatability and consistency.

**Figure 7:**
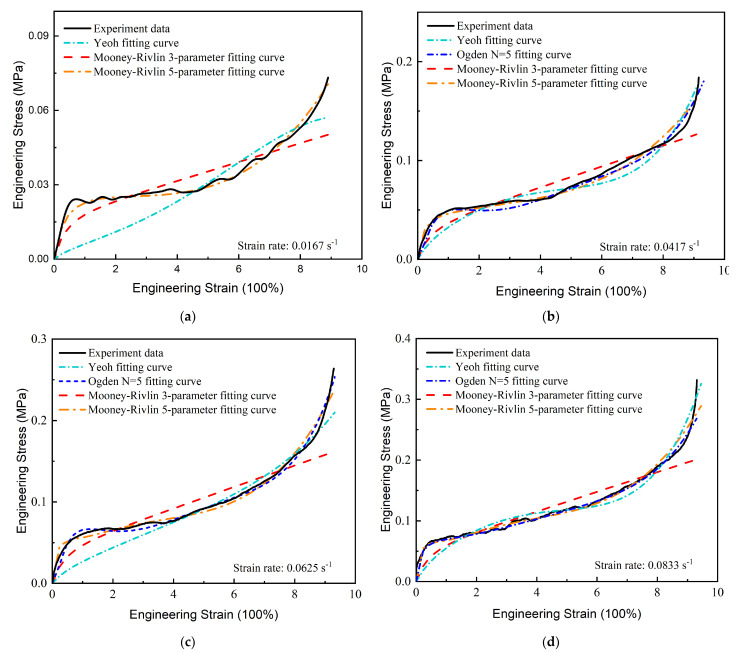
Fitting results of different constitutive models: (**a**) strain rate is set at 0.0167 s^−1^; (**b**) strain rate is set at 0.0417 s^−1^; (**c**) strain rate is set at 0.0625 s^−1^; (**d**) strain rate is set at 0.0833 s^−1^.

In this work, the three-parameter and five-parameter Mooney–Rivlin, Yeoh, and Ogden models, based on the phenomenological theory, were used to characterize hyperelastic properties. The fitting results under different strain energy density functions were obtained, as shown in Figure 7.

It can be seen that the 3-parameter Mooney–Rivlin model exhibited a poor fitting effect on the nominal stress–strain curve at different strain rates, while the 5-parameter model fit well across the entire deformation stage. The Yeoh model presented well with medium and large deformation (Figure 7b–d). The Ogden model was very flexible and the 5th order model fit well across the entire deformation stage when the strain rate was higher than 0.0417 s^−1^, but the Ogden model did not converge when the strain rate was set at 0.0167 s^−1^. The constitutive model with high-order was more complex in engineering application. Therefore, the 5-parameter Mooney–Rivlin model was selected to describe the hyperelastic properties. The relative fitting parameters are shown in Table 2.

### 4.2. Static Viscoelasticity of OCA Material

The stress–relaxation tests were carried out at a strain of 50% for 10 min in tensile mode on the OCA samples and the changes in stress and relaxation modulus with time were recorded. The intrinsic non-linear behavior of stress relaxation is defined by the reorganization of the internal structure after deformation. The molecular chain is in an extended state that is unbalanced and unstable when stretched, and it trends back to the curled state because of the molecule’s thermal motion. The restriction of crosslinks and interactions prevents the molecular chain from disentangling, as the strain is fixed, and the molecular chain slips along the direction of the external force so that the internal stress gradually decreases and finally relaxes to the equilibrium value.

**Figure 8:**
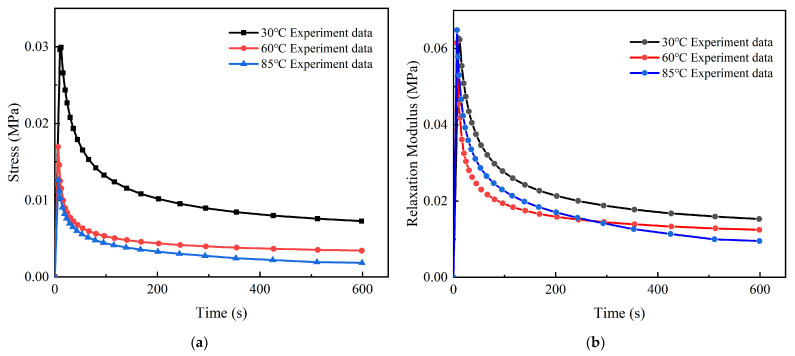
Stress evolution and relaxation modulus evolution: (**a**) stress evolution with time at different temperatures (30 °C, 60 °C, and 85 °C); (**b**) relaxation modulus evolution with time at different temperatures.

Figure 8 shows that both the stress and relaxation modulus rose rapidly in a very short time before reaching the specified strain, then decreased with time during the relaxation period and finally achieved a constant value. When the stress relaxation was carried out at 85 °C, the maximum stress was approximately 12.65 kPa. The initial stress decreased rapidly and finally reached a stable value of 1.84 kPa. The relaxation modulus decreased from 64.80 kPa to 9.51 kPa during the same process.

It can be seen that viscoelasticity was greatly affected by temperature. The temperature rise enhanced the mobility of the molecular chain segments and reduced the internal friction, which caused faster stress relaxation, lower stress, and a lower relaxation modulus at equilibrium.

**Figure 9:**
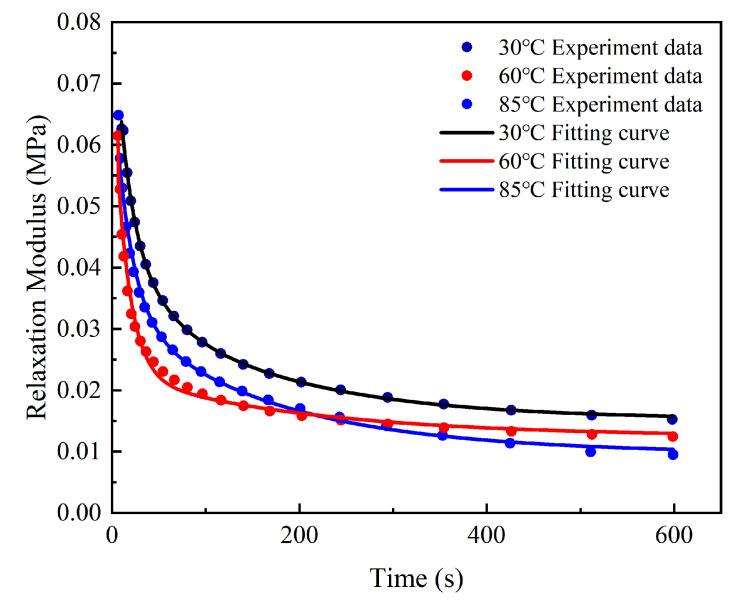
Fitting results of the generalized Maxwell model at different temperatures.

The generalized Maxwell model was fitted to the relaxation response to find the appropriate constitutive relationship, as shown in Figure 9. It can be seen that the 2nd order Maxwell model fit the experimental data at 60 °C well, while the 3rd order equation achieved a better fitting effect at 30 °C and 85 °C. The constitutive model curves matched well, with the R^2^ values exceeding 0.97. Table 3 illustrates the Prony coefficients for the OCA material at different temperatures (30 °C, 60 °C, and 85 °C).

### 4.3. Dynamic Viscoelasticity of OCA Material

**Figure 10:**
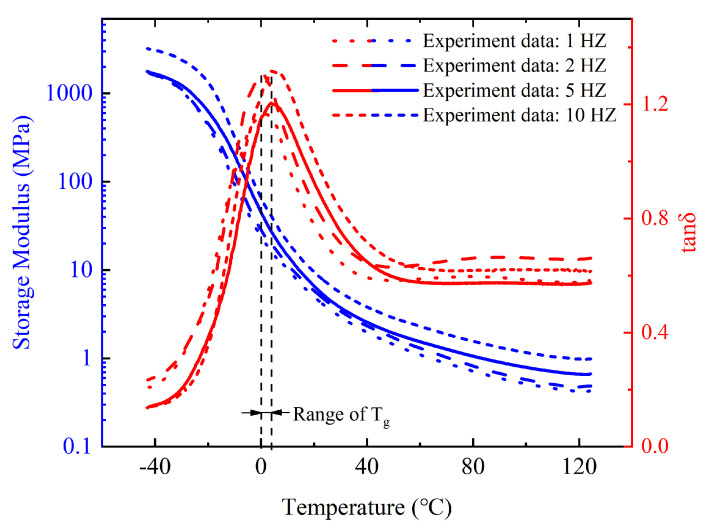
Variation of the dynamic modulus and the loss tangent with temperature at different frequencies.

As the viscoelasticity of OCA material is greatly dependent on the temperature, dynamic experiments of temperature sweeping are needed to better characterize the mechanical properties. The typical DMA results tested at different frequencies are shown in Figure 10. The storage modulus decreased continuously with the increase in temperature and decreased fastest around 0 °C (Figure 10). The rise in temperature intensified the thermal motion of molecules, resulting in a storage modulus decrease from the macroscopical view. When the temperature ranged from –40 °C to 125 °C at 5 Hz, the storage modulus decreased from the initial 1666.3 MPa to 0.669 MPa.

The loss tangent is defined as the ratio of loss modulus to storage modulus, and it exhibited a peak at around 0 °C in this experiment. Usually, the peak of loss tangent corresponds to the glass transition temperature (*T_g_*). Therefore, 0 °C was adopted as the *T_g_* of OCA material in our study. The molecular chain segments were in a frozen state with fixed positions when the material was below the glass transition temperature. The thermal energy was insufficient to surmount the potential barriers for translational and rotational motions of the polymer molecules segments within the state.

The OCA material changed from the glassy state to the rubber state as the temperature increased and the storage modulus decreased by four orders of magnitude during this process. It can be seen that viscoelasticity was greatly affected by temperature. In addition, the increase in loading frequency also led to an increase in storage modulus. The effect of temperature and frequency on dynamic viscoelasticity can be equivalent under certain conditions.

**Figure 11:**
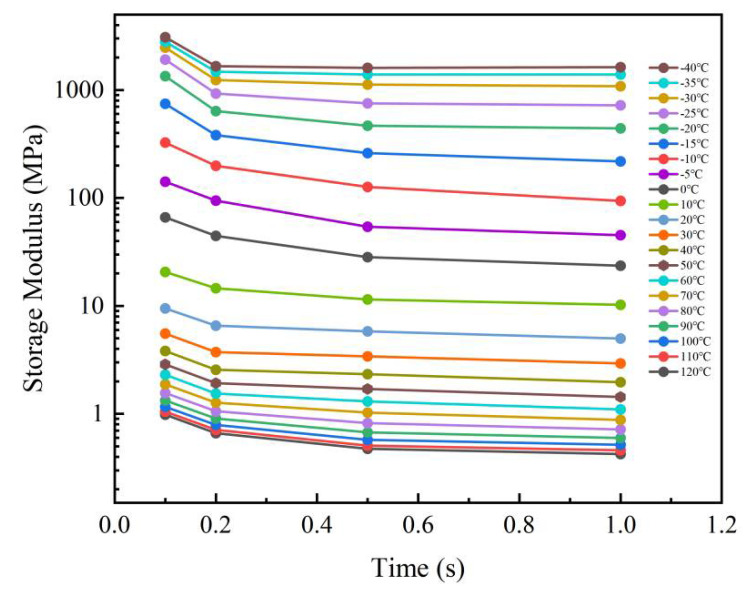
Storage moduli of OCA material at different temperatures.

Assuming that the OCA is thermorheologically simple, the storage modulus curves can be obtained by converting frequency coordinates to time coordinates at different temperatures. The DMA dynamic experiment results at different temperatures are as shown in Figure 11, where the storage modulus presents a decreasing trend over time. When the temperature was away from the 0 °C side, the change rate of storage modulus was smaller. The curves at different temperatures can be shifted according to the time–temperature superposition principle [32]. Following this, the master curve at a reference temperature can be generated.

**Figure 12:**
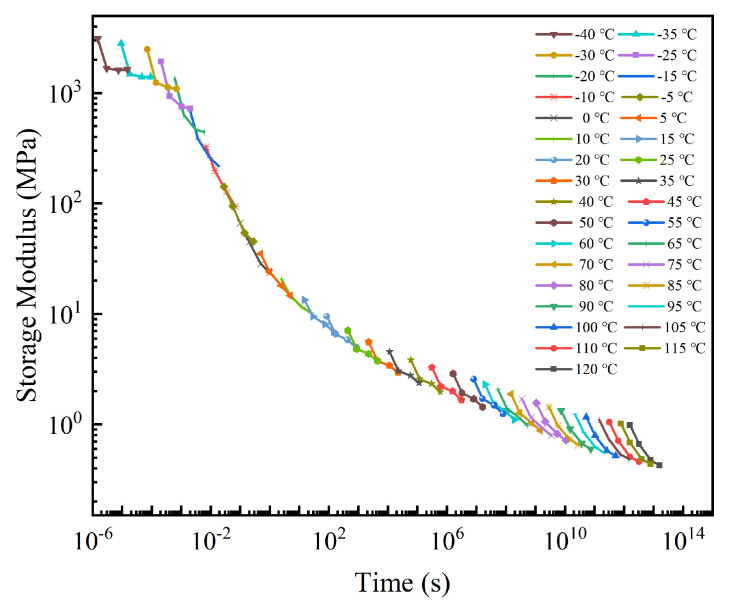
Master curve of the storage modulus (*T_ref_* = 0 °C).

In this work, the glass transition temperature (*T_ref_* = 0 °C) was selected as a reference. Then, the storage modulus curves at lower temperatures in were shifted horizontally to the left and the others shifted to the right (Figure 11). Finally, the master curve at 0 °C was obtained, as shown in Figure 12.

The obtained master curve of storage modulus was relatively smooth overall, and the time span can be extended to more than ten orders of magnitude greater than the experimental duration, showing a strong data retention ability.

**Figure 13:**
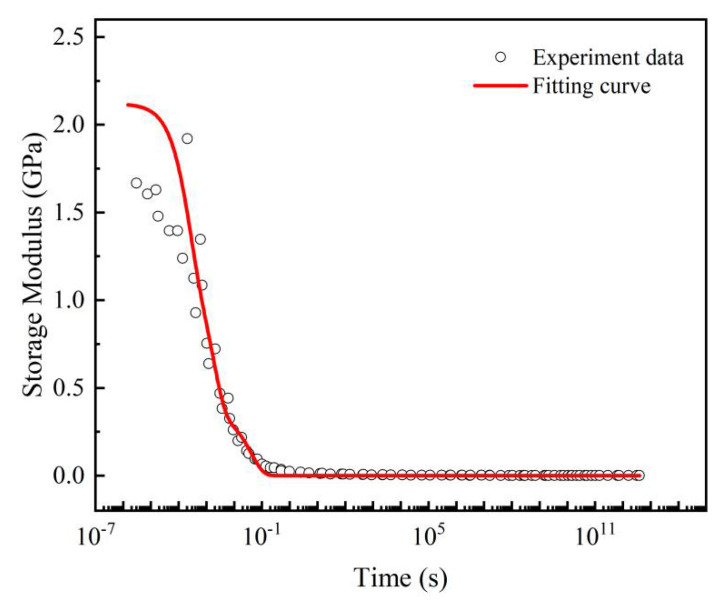
Generalized Maxwell model fitting for the master curve at 0 °C.

The generalized Maxwell model of the 3rd Prony series was used to fit the experimental data and characterized well, as shown in Figure 13. The fitting parameters are listed in Table 4.

**Figure 14:**
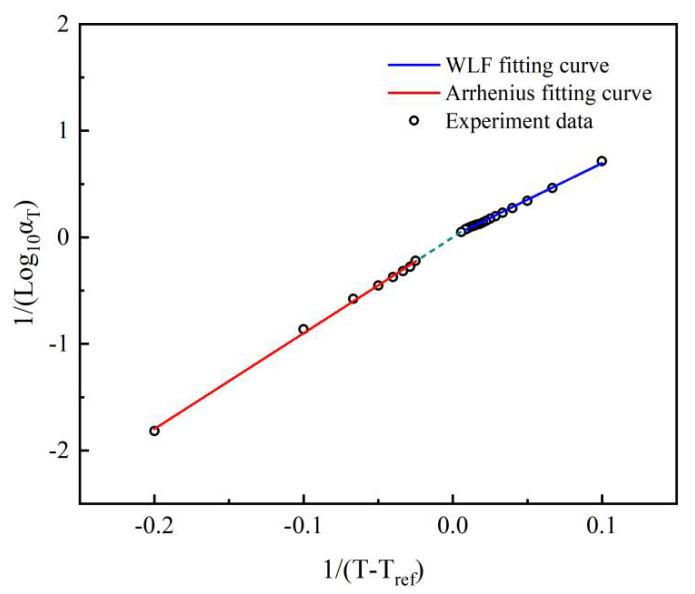
Shift factors for the master curve and the fitting results, *T_ref_* = 0 °C.

The corresponding shift factors, as shown in Figure 14, exhibited two different trends above and below the glass transition temperature. The free volume cannot be further reduced when the temperature is lower than the glass transition temperature, meaning that the WLF equation is no longer applicable. Therefore, two functions, the Williams–Landel–Ferry (WLF) equation and the Arrhenius equation, were used for modeling the temperature shift factors of the higher and the lower temperature, respectively. The expression can be written as:

(15) $$\frac{1}{{log}_{10}\alpha_{T}}=\{\begin{array}{cc} -\frac{C_{2}+\left(T-T_{ref}\right)}{C_{1}\left(T-T_{ref}\right)} & T>273\;K\\ \frac{R\cdot log_{e}^{10}}{\delta H}\cdot {\left(\frac{1}{T+273}-\frac{1}{T_{ref}+273}\right)}^{-1}=k\cdot \frac{1}{T}+b & T<273\;K \end{array}$$

where the *C*_1_ and *C*_2_ are the model constants of the WLF equation. *T_g_* = 0 °C is selected as the reference temperature. *R* = 8.314 J∙mol^–1^∙k^–1^ and *δH* is the material activation energy. The model constants are given in Table 5.

## 5. Conclusions

In this work, the hyperelastic and viscoelastic mechanical properties of OCA material were studied comprehensively. The hyperelasticity tests were conducted using the universal testing machine, and the nominal stress–strain curves obtained indicate the change in the material mechanical state across the entire tensile process. The Mooney–Rivlin, Yeoh, and Ogden models based on the phenomenological theory were used to characterize the hyperelastic properties. We found that the five-parameter Mooney–Rivlin and the third-order Ogden model both were very flexible and fit well across the entire deformation stage.

Furthermore, the DMA analyzer was applied to characterize the viscoelastic mechanical properties, and the viscoelasticity was greatly affected by temperature. The temperature rise enhanced the mobility of the molecular chain segments and reduced the internal friction, which caused lower stress and relaxation modulus at equilibrium. The initial stress at 85 °C was approximately 12.6 kPa, which was 57.4% lower than that at 30 °C and 25.66% lower than that at 60 °C, while the equilibrium relaxation modulus was approximately 9.51 kPa, 37.72% lower than that at 30 °C and 23.61% lower than that at 60 °C. In the viscoelastic dynamic test, the storage modulus monotonically decreased from 1666.3 MPa to 0.6628 MPa as the scanning temperature rose, and the decline rate reached the maximum near the glass transition temperature (*T_g_* = 0 °C). We found that the generalized Maxwell model of the 3rd Prony series characterized the data of the master curve very well. Two functions, the WLF equation and the Arrhenius equation, were used for modeling the temperature shift factors of the higher and the lower temperature, respectively, and both showed a good fit.

The test data and constitutive models can be used as design references in the manufacturing process, as well as for product reliability evaluation.

## Figures and Tables

**Table 1:** The parameters of test samples.

ID	Length (mm)	Width (mm)	Thickness (mm)	Length to Width Ratio
1	40	10	1	4:1
2	40	5	1	8:1
3	50	5	1	10:1

**Table 2:** Mooney–Rivlin model parameter values derived from tension test results.

Strain Rate/s^−1^	Order	Fitting Parameters			
0.0167	5-parameter	C_10_ = −1.357 × 10^−4^ C_20_ = 3.454 × 10^−5^	C_01_ = 1.302 × 10^−2^ C_11_ = −2.053 × 10^−4^	C_02_ = 3.639 × 10^−4^	D_1_ = 0 D_2_ = 0
0.0417	5-parameter	C_10_ = −4.258 × 10^−2^ C_20_ = 7.826 × 10^−5^	C_01_ = 8.166 × 10^−2^ C_11_ = −6.182 × 10^−4^	C_02_ = 1.284 × 10^−2^	D_1_ = 0 D_2_ = 0
0.0625	5-parameter	C_10_ = −0.1214 C_20_ = 1.966 × 10^−4^	C_01_ = 0.1885 C_11_ = −1.961 × 10^−3^	C_02_ = 3.749 × 10^−2^	D_1_ = 0 D_2_ = 0
0.0833	5-parameter	C_10_ = −0.1616 C_20_ = 2.270 × 10^−4^	C_01_ = 0.2463 C_11_ = −2.385 × 10^−3^	C_02_ = 4.976 × 10^−2^	D_1_ = 0 D_2_ = 0

**Table 3:** Prony coefficients obtained from the generalized Maxwell model.

Temperature (°C)	*i*	1	2	3
30	*g_i_*	0.4248	0.2436	0.3315
	*τ_i_*	14.368	37.209	161.71
60	*g_i_*	0.8166	0.1666	-
	*τ_i_*	15.034	203.425	-
85	*g_i_*	0.6172	0.2256	0.1563
	*τ_i_*	16.281	118.25	236.26

**Table 4:** Prony coefficients of the master curve.

	*i*	1	2	3
Prony Series Parameters	*g_i_*	0.171	0.222	0.0713
	*τ_i_*	2.19 × 10^−4^	1.77 × 10^−3^	0.0910

**Table 5:** Relevant parameters for the shift functions.

**WLF**	***C*_1_**	***C*_2_**	**Residual Sum of Squares**
	−81.3	550.4	0.0014
**Arrhenius**	***k***	***b***	**Residual Sum of Squares**
	8.98	−0.00231	0.0032

## Data Availability

Data available on request, having regard to restrictions, e.g., privacy or ethical.

## References

[1] Chiu T.C., Yeh E.Y. (2018). Warpage simulation for the reconstituted wafer used in fan-out wafer level packaging. Microelectron. Reliab..

[2] Sarvar F., Hutt D.A., Whalley D.C. Application of adhesives in MEMS and MOEMS assembly: A review. Proceedings of the 2nd International IEEE Conference on Polymers and Adhesives in Microelectronics and Photonics.

[3] Priyadarshi A., Shimin L., Mhaisalkar S.G., Rajoo R., Wong E.H., Kripesh V., Namdas E.B. (2010). Characterization of optical properties of acrylate based adhesives exposed to different temperature conditions. J. Appl. Polym. Sci..

[4] Leo K. (2011). Organic light-emitting diodes: Efficient and flexible solution. Nat. Photonics.

[5] Usafsson H., Cao Y., Racy M., Kavr F., Hrvol A.J. (1992). Flexible light-emitting diodes made from soluble conducting polymers. Nature.

[6] Min S.H., Kang M.K., Kim I.Y., Kim H.S., Chang K.K. (2012). A study on flexible OLED employing cellulose paper as a substrate. Mol. Cryst. Liq. Cryst..

[7] Chwang A.B., Rothman M.A., Mao S.Y., Hewitt R.H., Weaver M.S., Silvernail J.A., Rajan K., Hack M., Brown J.J., Chu X. (2003). Thin film encapsulated flexible organic electroluminescent displays. Appl. Phys. Lett..

[8] Li L., Yu Z., Hu W.L., Chang C.H., Chen Q., Pei Q.B. (2011). Efficient flexible phosphorescent polymer light-emitting diodes based on silver nanowire-polymer composite electrode. Adv. Mater..

[9] Jin D.U., Kim T.W., Koo H.W., Stryakhilev D., Kim H.S., Seo S.J., Kim M.J., Min H.K., Chung H.K., Kim S.S. (2010). Highly robust flexible amoled display on plastic substrate with new structure. SID Int. Symp. Dig. Tech. Pap..

[10] Campbell C.J., Clapper J., Behling R.E., Erdogan B., Beagi H.Z., Abrahamson J.T., Everaerts A.I. (2017). P-198: Optically clear adhesives enabling foldable and flexible OLED displays. SID Int. Symp. Dig. Tech. Pap..

[11] Wang H., Deng X., Wu H., Pi A., Huang F. (2019). Investigating the dynamic mechanical behaviors of polyurea through experimentation and modeling. Def. Technol..

[12] Qian Z.F., Wang J.J., Yang J., Liu S. (1999). Visco-elastic-plastic properties and constitutive modeling of underfills. IEEE Trans. Compon. Packag. Technol..

[13] Fu X., Wang Z., Ma L., Zou Z., Zhang Z., Guan X. (2020). Temperature-Dependence of rubber hyperelasticity based on the eight-chain model. Polymers.

[14] Yeh M., Chang L., Cheng H., Wang P. (2014). Bending stress analysis of laminated foldable touch panel. Procedia Eng..

[15] Salmon F., Everaerts A., Campbell C., Pennington B., Erdogan-Haug B., Caldwell G. (2017). 64-1: Modeling the mechanical performance of a foldable display panel bonded by 3M optically clear adhesives. SID Int. Symp. Dig. Tech. Pap..

[16] Ha M.H., Choi J.K., Park B.M., Han K.Y. (2021). Highly flexible cover window using ultra-thin glass for foldable displays. J. Mech. Sci. Technol..

[17] Yunsik C., Chae G.S., Youn Y.O., Woo S., Shin S.K., Lee J. (2018). Optimal design of thickness and Young’s modulus of multi-layered foldable structure considering bending stress, neutral plane and delamination under 2.5 mm radius of curvature. Int. J. Precis. Eng. Manuf..

[18] Cheng A., Chen Y., Jin J., Su T. (2019). Study on mechanical behavior and effect of adhesive layers in foldable AMOLED display by finite element analysis. SID Symp. Dig. Tech. Pap..

[19] Nishimura M., Hishinuma M., Yamaguchi H., Murayama A. (2020). Quantitative evaluation of neutral-plane splitting in foldable displays using folding stiffness measurements and finite element method simulations. SID Symp. Dig. Tech. Pap..

[20] Ma L., Gu J. (2020). 3D bending simulation and mechanical properties of the OLED bending area. Open Phys..

[21] Suchocki C., Jemioo S. (2021). Polyconvex hyperelastic modeling of rubberlike materials. J. Braz. Soc. Mech. Sci. Eng..

[22] Huang Z.P. (2014). A novel constitutive formulation for rubberlike materials in thermoelasticity. J. Appl. Mech..

[23] Beda T., Chevalier Y. (2003). Hybrid continuum model for large elastic deformation of rubber. J. Appl. Phys..

[24] Thanakhun K., Puttapitukporn T. (2019). PDMS material models for anti-fouling surfaces using finite element method. Eng. J..

[25] Darijani H., Naghdabadi R. (2010). Hyperelastic materials behavior modeling using consistent strain energy density functions. Acta Mech..

[26] Yeoh O.H. (2012). Some forms of the strain energy function for rubber. Rubber Chem. Technol..

[27] Mao Y., Li Y., Chen Y., Miao Y., Deng Q., Niu W. (2015). Hyperelastic behavior of two rubber materials under quasistatic and dynamic compressive loadings-testing, modeling and application. Polimery.

[28] Dorfmann A., Ogden R.W. (2006). Nonlinear electroelastic deformations. J. Elast..

[29] Peng M., Xu Z. (2006). Research on nonlinear constitutive relationship of permanent deformation in asphalt pavements. Sci. China Ser. G Phys. Mech. Astron..

[30] Renaud F., Dion J., Chevallier G., Tawfiq I., Lemaire R. (2011). A new identification method of viscoelastic behavior: Application to the generalized Maxwell model. Mech. Syst. Signal Processing.

[31] Zhao B., Hu J., Chen W., Chen J., Jing Z. (2020). A nonlinear uniaxial stress-strain constitutive model for viscoelastic membrane materials. Polym. Test..

[32] Ghaffari S., Ng E.J., Ahn C.H., Yang Y., Wang S., Hong V.A., Kenny T.W. (2015). Accurate modeling of quality factor behavior of complex silicon MEMS resonators. J. Microelectromech. Syst..

